# Impact of union practices on labor relations in China: Institutional trust as a moderator

**DOI:** 10.3389/fpsyg.2022.944574

**Published:** 2022-08-30

**Authors:** Yuanling Li, Zhongliang Dai, Xiao Hu

**Affiliations:** ^1^School of Public Policy and Administration, Chongqing University, Chongqing, China; ^2^Labor Relations Department of Business School, Southwest University of Political Science and Law, Chongqing, China; ^3^Student Affair Office, Southwest University of Political Science and Law, Chongqing, China

**Keywords:** union practice, institutional trust, labor relations climate, Chinese unions, labor–management relations

## Abstract

The particularities of Chinese union practices in the private sector and their impacts on the labor relations climate have raised much controversy. This paper presents the findings of a study that analyzed data from 926 enterprises in Chongqing, China, through the lens of institutional trust. The study was designed to examine the influence of union practices on the labor relations climate at the enterprise level. Particular attention was paid to the possible moderator effect that both employee and management trust in unions had on the labor relations climate. We found that employee–union trust positively moderated the impact of union practice on the labor relations climate. However, if management–union trust exceeded employee–union trust, management–union trust weakened the moderator effect of employee–union trust. In other words, management–union trust negatively moderated employee–union trust. This article is organized as follows. In section “Introduction,” we introduce the institutions Chinese unions operate in, especially regarding disputes over the effects on the labor relations climate. In section ‘Theory and hypotheses,” we review the literature and develop the hypotheses. In section “Materials and methods”, we describe the data and method, and in section “Results,” we present the results of the model. Finally, in section “Discussion,” we discuss the implications for China’s union development and note the limitations of the study.

## Introduction

The activities of unions have an important impact on the labor relations climate. Chinese unions are different from unions in other countries in that they have what have been referred to as “Chinese characteristics” (Zhu et al., 2011; Ge, 2014; Chan and Hui, 2018). For example, Chinese unions are formed from top to bottom. They adhere to the leadership of the Communist Party and maintain the production order of enterprises, while charged with the safeguarding of the rights and interests of workers. According to the *Constitution of the Chinese Trade Unions,* Chinese unions serve as a bridge and link between the Party and workers and represent an important social pillar of the state power. Therefore, the ostensible role of unions is twofold: they safeguard the rights and interests of workers and also serve to improve business productivity (Chen, 2010; Friedman, 2012). These features of Chinese unions have influenced the ways that researchers examine the role of Chinese unions in the labor relations climate. Scholars have been divided on their views of union effectiveness in China. Scholars who have found unions to be ineffective maintain that the characteristics of the formation process of unions in China have created a conflict. Consequently, unions have been incapable of effectively safeguarding the legitimate rights and interests of employees (Heng, 2010; Zhu and Zhu, 2022). They have become “shell unions,” “pseudo unions” and “boss unions” (Kai and Brown, 2013). They did not protect employees’ rights and interests. Rather, they serve as a facade to create the illusion that workers were protected (Jiang and Yao, 2020). Their presence as such has not been conducive to the improvement of the labor relations climate. Scholars who have argued that the unions are effective note that the adjustment and optimization of China’s unions practices in recent years, as well as the dual functions of unions, have synergistic effects that effectively improve workers’ rights (Snape and Redman, 2012; Chan et al., 2017; Huang et al., 2021). Moreover, union contributions to employees’ welfare and productivity have had a positive impact (Yao and Zhong, 2013; Ge, 2014; Liu and Li, 2014). The Chinese government also has identified the importance of labor capital harmony. Unions have protected workers’ rights in response to the government’s concern for the need to improve the labor relations climate (Wu and Sun, 2014; Chung, 2016).

There has been no scholarly consensus on the labor unions’ impact on the labor relations climate. Researchers have approached the topic from a variety of disciplines, theories and methods with inconsistent and opposing conclusions. The reason for this situation could primarily be attributed to the duality of China’s unions’ functions. Chinese unions were swing between the functions of safeguarding the interests of the employer and protecting the rights and interests of employees. Therefore, the impacts of union practice on labor relations have proven different during different time periods, varied contexts of policy priorities, and distinct social situations. However, some of the disagreement also could be due to the limitations of the research conducted. Previous studies have provided meso-level analyses of the impacts of union practices on labor relations rather than an examination of these practices on a micro level (Chen, 2010; Luethje, 2014; Anwar and Sun, 2015; Valizade, 2018; Guo and Laroche, 2021). The two opposing arguments deal with the labor relations climate from the perspective of conflicts over rights and interests between enterprises and workers. Under the influence of the unique “dual function” of Chinese unions, this research approach will naturally draw contradictory conclusions at the macro and meso-levels. At the enterprise level, however, there are interest-based, rights-based, and affect-based conflicts in the Chinese context (Xi et al., 2021). This means that emotional factors among management, union representatives, and workers will affect the labor relations climate. This insight provides a foundation for the present study. Among these emotional factors, trust is undoubtedly the most noteworthy, and it has been investigated in previous studies (Mayer et al., 1995; Innocenti et al., 2011; Kougiannou et al., 2015; Ehlers, 2020; Refslund, 2021). This study, therefore, aims to reveal the relationship between union practices and the labor relations climate based on trust factors rather than rights and interests at the enterprise level.

**Figure 1:**
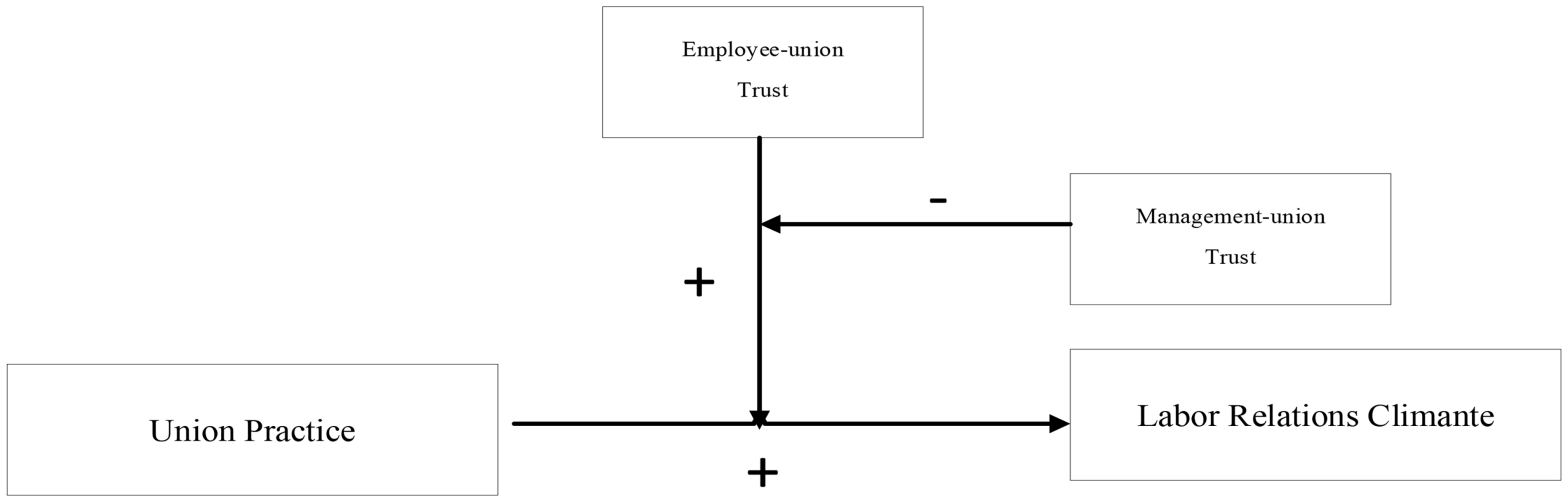
Proposed model.

To provide a better understanding of the role Chinese unions play in labor–management relations, we suggest that the relationship between union practices and the labor relations climate may be grasped based on the moderating role of institutional trust, including employee–union trust and management–union trust in labor unions. In addition, this research was designed to investigate the mechanism by which union practices affect the labor relations climate in situations involving varying levels of trust among employees, management, and unions. This approach also has implications for corresponding management countermeasures. Our research (summarized in Figure 1) aims to advance our understanding of the relationship between union practices and the labor relations climate in several ways. First, different from previous studies that focused primarily on regional union density and coverage (Yao and Zhong, 2013; Furaker, 2020; Budd and Lamare, 2021; Refslund, 2021; Ringqvist, 2021), this study extends from the macro level (national) to the meso level (regionals) and finally to the micro level (enterprises and employees). We collected data on the labor relations climate, employee–union trust, management–union trust, and union practice through surveys of employees, unions, and management at 926 enterprises. On that basis, we tested the relationship between union practice and the labor relations climate with institutional trust as a moderator. In this way, we enrich the research on the relationship between unions and labor–management relations in China. Second, by introducing the dimension of the legitimacy and effectiveness of institutional trust, we treated employee–union trust and management–union trust as specific mechanisms in which institutional trust plays a moderating role. Finally, based on the dual functions of Chinese unions, we identified situations in which union practice can effectively improve the labor relations climate, as well as situations in which trade union practices do not work.

## Theory and hypotheses

### Union practice and the labor relations climate

Union practice is a series of management activities that unions implement in enterprises. According to the *Constitution of the Chinese Trade Unions*, union functions include four aspects: service and maintenance, education and training, participation, and construction. Previous studies have shown that these four types of functions could be divided into the safeguarding of the labor rights and interests of employees and assistance for enterprises to perform management functions(Cooke, 2014; Chan et al., 2017). The latter protects the interests of employees without damaging business production and operations (Lee et al., 2016). However, others have argued that maintenance, care, participation, promotion, and construction constitute the main dimensions of union practices and that Chinese unions have a “triple-face”: a strong “State-Party voice” face, a weak “monopoly” face, and a significant “collective voice” face (Ge, 2014). Although these definitions of union practice have differed, there has been agreement that union practice is distinct from human resource management activities. They also have coincided in their emphasis on employee participation, employee care, and the protection of employee rights and interests.

The labor relations climate is a subset of the organizational climate, which refers to a set of variables representing the norms, feelings, and attitudes prevailing in a workplace (Payne and Mansfield, 1978). The labor relations climate originates from labor relations activities and is perceived by organizational members (Dastmalchian et al., 1989). Therefore, viewed in this way, the concept of the labor relations climate is perceptual rather than objective and is “organizational” rather than “psychological” (Jones and James, 1979). Given the decreases in union coverage and increases in non-union workplaces, researchers have recognized that the labor relations climate is a multidimensional concept that is related to more than just labor relations (Barrett, 1995; Buchanan, 2008). Thus, the labor relations climate refers to the atmosphere, norms, attitudes, and behaviors reflecting and underpinning how workers, unions, and managers collectively interact with each other in the workplace, which in turn, affects workplace outcomes (Pyman et al., 2010). This conclusion also supports the view that labor relations are a function of the interactions between organizational members (Schneider and Reichers, 1983).

The labor relations climate is similar to an organizational climate, but unique in that it is generated by both labor and capital. Chan et al. (2004) counter the suggestion that the labor relations climate primarily involves differences between enterprises and unions (Fuller and Hester, 1998), by noting that the climate refers to a psychological feeling of employees about the relationship between themselves and enterprises (Chan et al., 2004). This study also was based on the understanding that the labor relations climate has psychological underpinnings. The labor relations climate impacts levels of cooperation from employees, union representatives, and business managers in the workplace, and the quality of operations throughout the entire organization (Pyman et al., 2010). Therefore, further research on the labor relations climate in the workplace proves crucial, especially with regard to the role of labor unions (Dastmalchian et al., 1989; Jiang and Yao, 2020). First, in situations with strong capital and weak labor, a business’ reliance on compulsory power often has led to employee dissatisfaction, making conflict resolution a contentious process (Murphy, 2020). However, when unions have participated in the implementation of business practices, there has been an improved labor relations climate (Newman et al., 2019). Second, cultural, artistic, recreational, athletic, and competitive activities organized by Chinese unions have helped to encourage a greater sharing of interests and job satisfaction (Hu et al., 2018). Finally, union initiatives have added instrumental and emotional value to the labor relations (Sverke and Kuruvilla, 1995; Mellor and Golay, 2014; Redman and Snape, 2014). Unions have helped to balance the conflicting interests between labor and capital, through their work to improve the wages, benefits, and working conditions of employees (Xie and Zhou, 2020). Labor union initiatives contribute to emotional well-being through efforts that result in employee recognition and awareness of their importance. Therefore, we propose our first hypothesis:

*H1*: Union practice improves the labor relations climate.

### Trust of employees and management in unions

The labor relations climate originates from the interactive relationships among employees, management, and union representatives. Therefore, the matter of trust proves crucial to scholarly work on labor relations. Trust is built through a dynamic process (Tyler, 2003). People have been most willing to take the risk of trusting someone else based on positive expectations of the other’s intentions or behaviors (Angelovski et al., 2019). When levels of trust are low, a subject would not have positive expectation of the other’s actions. Moreover, they could anticipate that their own interests would be compromised and engage in risk avoidance behaviors. Therefore, the level of trust status and relationships would have a significant impact on their actions (Balliet and Van Lange, 2013).

Trust in unions typically has been treated as institutional trust (Shapiro, 1987). One competitive advantage of institutional trust lies in persistence (Vanhala et al., 2011). Even when interpersonal trust has been challenged, institutional trust could persist. Institutional trust could be separated into the spheres of content and function. A person’s function within the institution function would directly affect their trust in the system itself (Farrell and Knight, 2003). Institutional trust emerges from a subject’s judgment of the purpose and value of the system; their perception of the system’s legitimacy would affect institutional trust. This trust also is grounded in the effect of the system’s operations. The system’s effective implementation, coupled with the achievement of objectives, also impact institutional trust. The trust of employees in the union (employee–union trust) has been evidenced primarily by the extent to which employees believe that the practice of the union is to protect their own rights and interests. Similarly, management trust in the union (management–union trust) exists when management treats union practices as conducive to the interests of enterprises (Ehlers, 2020).

Investigations into employee–union trust and management–union trust through the dimensions of legitimacy and effectiveness have revealed that the bases for the two types of institutional trust were not consistent. Because unions have strong labor attributes, they should naturally represent and safeguard labor rights and interests. Therefore, employees’ trust in unions has been established first on a cognitive basis; employees believe that unions perform their duties to protect employee rights and interests, similar to institutional trust in the dimension of legitimacy. In China, unions have been charged with the functions of “organizing employees, guiding employees, serving employees, and safeguarding the legitimate rights and interests of employees” (Huang et al., 2016). They have served to provide members with a sense of identity and have a broad employee base. Therefore, the foundation of employee–union trust has emerged primarily from the dimension of legitimacy.

The union as the employee representative should have independence. However, because unions are funded by 2% of workers’ wages deducted from paychecks by the business according to the *Constitution of the Chinese Trade Unions*, they also are economically dependent on the enterprise. Therefore, the source of trust in unions for employees and management differs: employee–union trust has relied heavily on legitimacy, whereas management–union trust has been based on effectiveness. The union’s interests necessarily were tied to the business, eliminating the need for legitimacy. Management typically has viewed the labor union as one of the business’ many departments or units. Once the legitimacy dimension has been eliminated, the effectiveness dimension of institutional trust becomes particularly important. Management has devoted attention to the functions of unions for employee training and service production, because those activities contribute to business performance (Huang et al., 2021). Therefore, management–union trust has been shaped by the effectiveness of union efforts for enterprise performance. In short, although employee–union trust and management–union trust both form part of institutional trust, their sources are not the same. This key difference has played a vital role in the impact of union practices on labor–management relations.

### Moderating effect of employee–union trust and management–union trust

Trust has always been regarded as a positive factor in the organization and can effectively support the expectation of results (Butler, 1991). Sufficient employee–union trust may encourage employees to have a strong sense of identity with union activities and may actively engage in and evaluate those efforts (Xie and Zhou, 2020). The union activities would be regarded by employees as actions to safeguard their rights and interests as well as an expected source of organizational support (Kougiannou et al., 2015). Furthermore, employees have been more likely to feel cared for by the employer when there has existed employee–union trust, resulting in greater job satisfaction (Wei et al., 2021). Employee–union trust has been shown to correlate with emotional and normative commitment to an organization (Lewicka, 2020). The promotion of emotional commitment has helped to ensure that employees devote themselves to their work and improve organizational citizenship. The promotion of normative commitment has encouraged employees’ loyalty to the enterprise and enhanced employees’ sense of responsibility to the enterprise (Sverke and Kuruvilla, 1995; Yuan et al., 2014; Ocasio and Radoynovska, 2016). Both of these aspects have been shown to improve the labor relations climate. Therefore, employee–union trust has contributed to a positive relationship between employees and management, increased willingness on the part of employees to actively serve the enterprise and take initiative on the creation of a harmonious work environment, and improved labor–management relations. From this, we draw the following hypothesis:

*H2*: Employee–union trust conditions the relationship between union practices and labor relations. When employee–union trust is high, union practice plays a greater role in improving the labor relations climate. When that trust is low, union practice has little or no effect on the improvement of labor relations climate.

The complexity of the relationships between employees and management emerges primarily from the interactions between the legitimacy and effectiveness dimensions of institutional trust; the role of employee–union trust is subject to management–union trust. The effectiveness dimension of institutional trust could strengthen or weaken the legitimacy dimension. Therefore, the practical effect of unions should be examined, especially with regard to the protection of workers’ rights and interests. Management–union trust results primarily from the effectiveness dimension of the system. When trust between the two has been strong, the union acted to further business interests, helping the enterprise complete production tasks and educating workers to support the enterprise. Chinese unions have always proved lacking in the performance of functions that protect employees’ rights and interests and attend to employees’ participation within the enterprise. This situation weakens the employee–union trust in the dimension of legitimacy.

A union’s decision to prioritize one function over another could create tension between employee–union trust, derived mainly from the legitimacy dimension, and management–union trust, derived mainly from the effectiveness dimension of institutional trust. Two possible scenarios could ensue. First, the prioritization of employee–union trust would result in a strengthening of employee commitment and union practice would improve the labor relations climate. However, if employees perceived that the fundamental purpose of the union was to serve the interests of the enterprise rather than protect their own rights and interests, the legitimacy dimension of institutional trust between the two could be weakened or even lost. Management–union trust also would impact the moderating effect of employee–union trust on the relationship between union practice and the labor relations climate. Based on these observations, we propose the following hypotheses:

*H3a*: When management–union trust exceeds employee–union trust, the stronger the management–union trust, the weaker the moderating effect of employee–union trust on union practice and the labor relations climate.

When employee–union trust exceeds management–union trust, the re-moderating effect of management–union trust will no longer exist because the tension between the legitimacy and effectiveness dimensions in institutional trust will be reduced. Therefore, we also propose another hypothesis:

*H3b*: When employee–union trust exceeds management–union trust, management–union trust will not affect the moderating effect of employee–union trust on union practice and labor relations climate.

## Materials and methods

### Data collection and sample

In cooperation with the Chongqing Municipal Human Resources and Social Security Bureau, the research team collected data from 1,140 enterprises in 26 districts and 12 counties of Chongqing, Southwest China, from August to December 2019. The specific sample selection and questionnaire distribution procedures included several steps. First, we randomly selected 30 enterprises from each district and county that have established unions and recruited participants from each of those businesses. We contacted a senior manager at each enterprise, asking for approval to administer the survey, and discussing the schedule. Next, we went to each enterprise. With the assistance of the enterprises’ human resources department and union office, respondents were asked to complete three types of questionnaires. The first was a management questionnaire about the trust of the management in the union, which was completed by an executive familiar with the enterprise, such as the founder, chairman, general manager, or human resources director. The second was an employee questionnaire about the labor relations climate and employees’ trust in the union, which was administered to 10 employees at each site. All qualified employees available that day were informed of the survey and invited to participate on a voluntary basis. The survey was conducted in a quiet meeting room near the respondents’ work site. An average of the responses was obtained. The third was a union questionnaire about union work and activities, which was completed by the union president or vice-president. A total of 926 groups of valid responses were collected; each group was a set consisting of management, employees, and unions. The recovery rate was 81.2%.

A total of 196 of these firms were located in the main urban area of Chongqing, 234 in the northeast area of Chongqing, 380 in the western area of Chongqing, and 126 were from the southeast area of Chongqing; A total of 368 (38.2%) enterprises were from the manufacturing industry, and the others were distributed among 11 industries: high-tech, IT, finance, real estate, retail, construction, hospitality and catering, business and leasing services, transportation, warehouse, and delivery services. The businesses included had an average number of 289.6 employees and had been in business an average of 14 years. Among the employees who filled out the questionnaire, 52.3% were male and 47.7% were female. A little over one-third of them (36.9%) had a high school education or below, and 5,866 people (61.0%) had a college education. Only 90 (<1%) of the participants had a graduate education. Most of the employees (52.6%) were between the ages of 30 and 39. Participants between the ages of 40 and 49 accounted for approximately one-third (32.9%) of the sample. Only 8% of the participants were over 50 years of age and 3% were under the age of 30. The participants had worked an average of 6.4 years for their current employer, for an average monthly income of RMB 4261. The vast majority (85.8%) were permanent employees.

### Measures

Some scales in our study were constructed in English, translated into Chinese by a management researcher, and then back-translated into English by another scholar. Two management professors who were fluent in both Mandarin and English were invited to make further modifications to these translations to enhance accuracy. All items were assessed using five-point Likert scales (1 = strongly disagree, 5 = strongly agree).

Union practice (UP) was determined using a 12-item scale in the union questionnaire that had been previously developed and validated by Chinese scholars (Zhang et al., 2018). Items included some of the following: “facilitates the life of employees,” “participates in the formulation and implementation of enterprise rules and regulations on behalf of employees,” and “organizes interesting competitions and activities that involve work skills.” The chairman or vice-chairman of the union responded to the items; the Cronbach’s α for the single index measure was 0.976.

Employee–union trust (ET) and management–union trust (MT) were captured using an eight-item employee questionnaire and management questionnaire developed and validated by Ellonen et al. (2008). Items included the following: “union activities serve our interests,” “relevant union systems have been well implemented,” and “the union can keep its promise.” These items were used to measure the trust of employees and management in unions. Employees and enterprise representatives responded to the items; the Cronbach’s α for the single index measure was 0.918.

Labor relations climate (LR) was measured using a 17-item employee questionnaire developed and validated by Cui et al. (2012). This measure was chosen for two reasons: First, the measure was designed within the Chinese context and therefore represented the current realities of present-day China. Second, the measure reflected employees’ subjective judgment of the relationship between the enterprise and the employees, meeting a study requirement. Sample items included “there is a harmonious climate in the company,” “the relationship between management and employees is harmonious,” “management and employees in this company can trust each other,” and “it is OK for me to work over time for a better future of the company.” Employees responded to the items; the Cronbach’s α for the single index measure was 0.977.

We controlled position, gender, marital status, age, education, salary, work years, and employment type at the individual level. We also controlled industry, location, firm age, and number of employees at the firm level, because these firm characteristics have a contextual effect on the labor relations climate.

The descriptive statistics of the main variables and control variables are shown in Table 1. The minimum value of union practice was 1, the maximum value was 5, and the average value was 4.67. The average value of the labor relations climate was 4.58. The average value of employee–union trust was 4.57, and the average value of management–union trust was 4.76. Table 2 shows the correlation coefficients for all variables. There was a positive correlation between union practice and the labor relations climate, employee–union trust and the labor relations climate, and management–union trust and labor relations climate.

**Table 1:** Statistical description of variables.

Variables	Mean	SD	Min	Max
UP	4.67	0.625	1	5
LR	4.58	0.316	2.30	5
ET	4.57	0.496	2.18	5
MT	4.76	0.541	1	5
Position	3.27	1.084	1	6
Gender	0.51	0.234	0	1
Marital status	1.87	0.167	1	2
Age	1.48	0.413	1	4
Education	1.89	0.554	1	3.4
Salary	4279.72	1295.10	1,800	12,450
Work years	6.52	4.585	0.8	30
Employment type	3.72	0.563	1	4
Industry	6.29	3.781	1	12
Location	2.45	0.960	1	4
Firm age	13.97	10.198	1	84
Number of Employees	289.64	759.255	120	13,799

UP, Union practice; LR, Labor relations climate; ET, Employ–union trust; MT, Management–union trust. Gender is coded 1 for male and 0 for female. Marital status is coded 1 for unmarried and 2 for married. Age is coded 1, 2, 3, and 4 for under 30, 30–39, 40–49, and over 50, respectively. Location is coded 1–4 for the four different regions of Chongqing. Industry is coded 1–12 for 12 different industries.

**Table 2:** Correlations and reliability of the variables.

	1	2	3	4	5	6	7	8	9	10	11	12	13 t	14	15	16
LR	1															
UP	0.65^*^	1														
ET	0.43^*^	0.44^*^	1													
MT	0.37^*^	0.31^*^	0.31^*^	1												
Position	−0.04	−0.07^*^	0.04	0.07^*^	1											
Gender	−0.00	0.01	−0.07^*^	0.00	−0.37^*^	1										
Marital status	−0.04	−0.02	−0.04	0.03	−0.11^*^	0.03	1									
Age	−0.06^*^	−0.04	−0.10^*^	−0.06^*^	−0.21^*^	0.26^*^	0.44^*^	1								
Education	0.11^*^	0.07^*^	0.12^*^	0.08^*^	0.26^*^	−0.11^*^	−0.38^*^	−0.43^*^	1							
Work years	0.11^*^	0.10^*^	0.01	0.05	−0.04	0.04	0.21^*^	0.29^*^	0.10^*^	1						
Employment type	0.16^*^	0.15^*^	0.04	0.07^*^	−0.09^*^	0.04	0.03	−0.09^*^	0.10^*^	0.16^*^	1					
Industry	−0.03	−0.07^*^	−0.02	0.11^*^	0.29^*^	−0.10^*^	−0.03	0.04	0.12^*^	0.00	−0.18^*^	1				
Location	−0.02	−0.01	−0.03	0.10^*^	0.06^*^	−0.01	0.12^*^	0.04	−0.09^*^	0.02	−0.03	0.08^*^	1			
Firm age	0.09^*^	0.09^*^	0.03	−0.01	−0.04	0.01	0.09^*^	0.12^*^	0.01	0.51^*^	0.10^*^	−0.09^*^	−0.09^*^	1		
Salary	0.08^*^	0.03	0.04	0.09^*^	−0.16^*^	0.20^*^	−0.08^*^	−0.09^*^	0.36^*^	0.15^*^	0.14^*^	−0.12^*^	−0.15^*^	0.08^*^	1	
Number of employees	0.09^*^	0.07^*^	0.07^*^	−0.04	−0.05	−0.01	−0.06^*^	−0.10^*^	0.13^*^	0.14^*^	0.08^*^	−0.16^*^	−0.14^*^	0.19^*^	0.13^*^	1

Firm age, salary, and number of employees were converted to a logarithmic scale. All variables were standardized.

* *p* < 0.10.

## Results

To test construct validity, confirmatory factor analysis (CFA) was conducted using scale items for the four study constructs (UP, ET, ME, LR). This CFA yielded an acceptable fit: *χ*^2^/d.f. = 2.1, CFI = 0.941, TLI = 0.951, RMSEA = 0.047, SRMR = 0.041. CFA that combined the two moderator variables, ET and MT, resulted in a poorer fit: *χ*^2^/d.f. =2.9, CFI = 0.752, TLI = 0.831, RMSEA = 0.071, SRMR = 0.053. Finally, a one-factor measurement model (all indicators loaded on a single factor), which is a variant of Harman’s single-factor test for common-method variance, resulted in a poorer fit: *χ*^2^/d.f. = 4.6, CFI = 0.582, TLI = 0.632, RMSEA = 0.064, SRMR = 0.112. Taken together, these results provide evidence for the construct validity of the measures used in this study.

We performed hierarchical multiple regression, gradually adding control variables, independent variables, and independent variable interaction items for analysis. The VIF of all variables was less than 5. Union practice was introduced first to test the impact of union practice on the labor relations climate. We then introduced the dual interaction between employee–union trust and union practice to test the moderating effect of employee–union trust. Finally, we introduced the interaction between management–union trust, union practice and employee–union to test the re-moderating effect of management–union trust. A total of 573 sets of responses were selected to test the re-moderating effect of management–union trust. The regression results are shown in Table 3. The *F* values of all of the models proved their significance. Our hypothesized model (as shown in Figure 1) with the controls included had good fit with the data: *χ*^2^/d.f. = 2.2, CFI = 0.941, TLI = 0.950, RMSEA = 0.046, SRMR = 0.041.

**Table 3:** Results of regression analysis.

	(1)	(2)	(3)	(4)	(5)
	Model 1	Model 2	Model 3	Model 4	Model 5
Position	−0.016	−0.001	−0.003	−0.006	−0.009
	(−1.41)	(−0.09)	(−0.34)	(−0.59)	(−0.67)
Gender	−0.013	−0.011	−0.002	0.007	−0.038
	(−0.26)	(−0.28)	(−0.06)	(0.16)	(−0.61)
Marital status	−0.046	−0.034	−0.043	0.014	−0.177^**^
	(−0.64)	(−0.61)	(−0.79)	(0.20)	(−1.99)
Age	−0.026	−0.012	−0.010	−0.005	−0.003
	(−0.82)	(−0.47)	(−0.39)	(−0.16)	(−0.07)
Education	0.038	0.013	0.003	0.027	−0.024
	(1.57)	(0.67)	(0.18)	(1.22)	(−0.74)
Work years	0.031	0.013	0.018	0.015	0.011
	(1.60)	(0.89)	(1.22)	(0.89)	(0.41)
Employment type	0.073^***^	0.031^**^	0.034^**^	0.019	0.062^**^
	(3.86)	(2.06)	(2.32)	(1.14)	(2.39)
Industry	0.001	0.003	0.003	−0.002	0.003
	(0.47)	(1.22)	(1.36)	(−0.66)	(0.86)
Location	0.004	0.001	0.002	−0.013	0.014
	(0.36)	(0.13)	(0.29)	(−1.31)	(0.96)
Firm age	0.017	0.005	0.004	−0.001	0.013
	(1.07)	(0.43)	(0.34)	(−0.08)	(0.59)
Salary	0.005	0.039	0.032	−0.031	0.063
	(0.13)	(1.18)	(0.99)	(−0.72)	(1.24)
Number of employees	0.014	0.009	0.008	0.012	0.009
	(1.61)	(1.26)	(1.19)	(1.56)	(0.73)
UP		0.319^***^	0.291^***^	0.318^***^	0.215^***^
		(24.64)	(19.22)	(8.24)	(6.61)
ET			0.119^***^	0.097	0.034
			(6.80)	(0.78)	(0.93)
UP×ET			0.031^**^	0.264^*^	−0.022
			(2.14)	(1.82)	(−0.75)
MT				0.092^***^	0.315^***^
				(2.90)	(3.46)
UP×MT				−0.068	0.044
				(−1.48)	(0.55)
ET × MT				−0.056	0.055
				(−0.58)	(0.77)
UP×ET × MT				−0.241^***^	−0.035
				(−2.59)	(−0.61)
_cons	0.000	0.000	−0.005	−0.002	−0.069^*^
	(0.00)	(0.00)	(−0.57)	(−0.06)	(−1.86)
N	926	926	926	573	353
r2	0.049	0.429	0.457	0.481	0.524
r2_a	0.04	0.42	0.45	0.46	0.50

Firm age, salary, and number of employees were converted to a logarithmic scale. All variables were standardized. Values in parentheses are t-values.

* *p* < 0.05,

** *p* < 0.01, and

*** *p* < 0.001.

**Figure 2:**
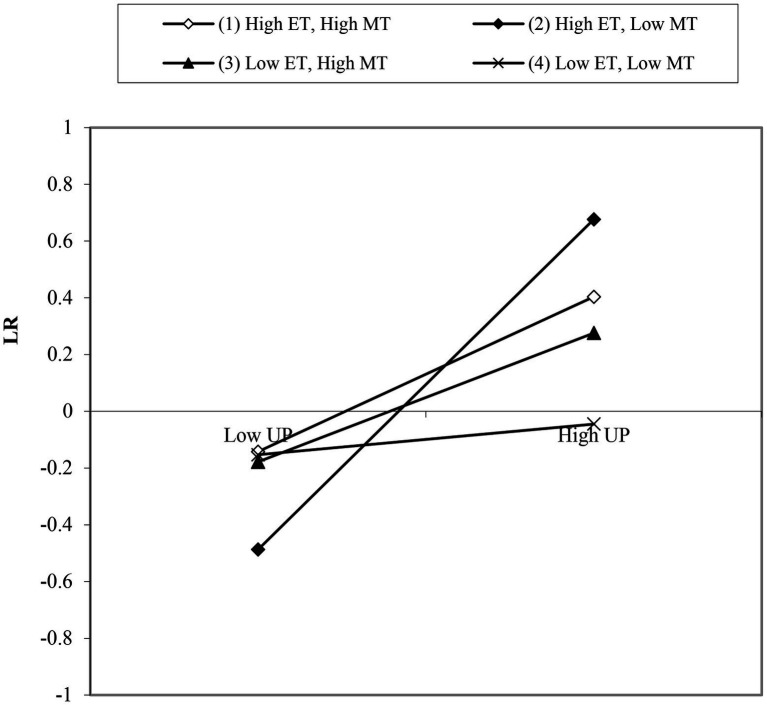
Moderating effects of employee–union trust and management–union trust.

Model 1 (Table 3) illustrates that with the control variables, only 4% of the variables in labor relations climate could be explained. Model 2 shows that union practice had a positive impact on the labor relations climate (*β* = 0.319, *p* < 0.01), which explained 42% of the variation in the labor relations climate. Therefore, hypothesis 1 was verified. The employee–union trust (ET) variable and its interaction with union practice were included in Model 3. The results indicated that union practice and its interaction with employee–union trust had a positive impact on the labor relations climate (*β* = 0.291, *p* < 0.01; *β* = 0.031, *p* < 0.05), and greater employee trust enhanced the impact of union practice on the labor relations climate. Figure 2 also confirms this conclusion. Therefore, hypothesis 2 was verified.

Model 4 tested the impact of union practice on the labor relations climate for a situation in which management–union trust exceeded employee–union trust, and it also was positive (*β* = 0.318, *p* < 0.01). The two interactions between union practice and employee–union trust were both positive (*β* = 0.264, *p* < 0.01). However, the interaction between union practice and employee–union trust and management–union trust was negative (*β* = −0.241, *p* < 0.01). This result showed that the inclusion of both employee–union and management–union trust revealed that stronger management–union trust resulted in a weakened moderating effect of employee–union trust on the relationship between union practice and the labor relations climate. Figure 2 also confirms this conclusion. Therefore, hypothesis 3a was verified.

The relationships between various variables in Model 5 were tested for a situation in which employee–union trust exceeded management–union trust. The results showed that the interaction coefficient of union practice, employee–union trust, and management–union trust was not significant. Therefore, in situations where employee–union trust was higher than management–union trust, management–union trust did not have a moderating effect. Thus, hypothesis 3b was verified.

To ensure that the results were not driven by other contextual factors, we ran additional analyses applying controls for location and replacing the number of employees with the *per capita* asset size of enterprises. The results were robust to these model specifications as well.

## Discussion

### Theoretical implications

Union practices impacted the labor relations climate. This research reaffirmed the findings from previous studies. The inclusion of trust to the study of union practice and the labor relations climate responds to scholarly concerns about the need to better understand the role of institutional trust in the field of enterprise management (Lewicka, 2020).

We found that employee–union trust had a positive moderating effect on both union practice and the labor relations climate. We also found that the interactions among union practice, employee–union trust, and management–union trust were significant for the situation in which employee–union trust exceeded management–union trust, indicating that management–union trust had a significant moderating effect. This shows that the role of trust between multiple subjects is complex.

This study explained the mechanisms of employee–union trust and management–union trust that impact the labor relations climate through union practice, drawing on theories of institutional trust. Employee–union trust based on the dimension of legitimacy and management–union trust in the dimension of effectiveness together shape the union practices that affect the labor relations climate. We showed that the effectiveness dimension of institutional trust weakened the influence of the legitimacy dimension. Management–union trust weakened the moderating effect of employee–union trust on the relationship between union practice and the labor relations climate in cases where management–union trust exceeded employee–union trust. However, in cases where that was not the case, there was no re-moderating effect of management–union trust; the legitimacy dimension of institutional trust was not significantly impacted by the effectiveness dimension. This study focused on trust and revealed the impact of union practice on the labor relations climate in different trust situations in order to contribute to the debate about the impact of union practice on the labor relations climate in China. We identified the shifts between the legitimacy dimension and effectiveness dimension of institutional trust. The identification of this mechanism could serve to expand and complement existing studies about the role of trust and offer an alternative perspective on the duality of union functions and their role in the labor relations climate. In other words, by analyzing different sources of trust and grasping the transformation mechanism of the legitimacy and effectiveness dimensions of institutional trust, we have clarified the situation in which union practices can improve the labor relations climate. This helps fill a gap in the existing research.

### Practical implications

This paper revealed the role of unions and the mechanism by which they could serve to improve the labor relations. There are several important implications of this research for enterprise management:

First, managers could strengthen the role of Chinese unions in the cultivation of labor relations. They could encourage enterprise unionization as a means by which to improve the labor relations climate. Second, managers who are conscious of the impact of the dual characteristics of China’s union functions could make decisions about the importance of unions for both production development and the safeguarding of workers’ rights and interests. Management promotion of both of these union roles could enhance the coordination of employee and business interests and create an institutional environment conducive to an improved labor relations climate. Finally, in view of the limited management resources available in some businesses, managers should focus on the factors that have the greatest impact on their objectives and attend to the trust between employees and enterprises and unions. For example, they could strengthen the legitimacy of the union through direct election of the president.

### Limitations and future research

This study was not designed to separately examine the distinct dimensions of union practice or to identify the specific impacts of those different dimensions on the labor relations climate. Therefore, future research on this topic could attend to the treatment of those distinct dimensions in order to identify the particularities of the multiple dimensions of union practice. This research focused on Chinese unions, which have a unique position in the country’s economy. Caution should be exercised in extending the relevancy of the findings to the labor relations climates in other settings.

## Data availability statement

The raw data supporting the conclusions of this article will be made available by the authors, without undue reservation.

## Author contributions

YL devised the research idea, developed the research model, performed the results, discussion, recommendations, limitations, revised and improved the introduction and method parts for the manuscript, and arranged the last version of the manuscript. ZD wrote the method part, ran the data collecting process ran the analytic calculations, limitations, implications, and checked for the literature and discussion part. XH wrote the introduction part and controlled the other parts in terms of language and contextual check for the manuscript. All authors contributed to the article and approved the submitted version.

## Funding

This work was supported by the Humanities and Social Sciences Research Programs of Chongqing Education Commission (21SKGH012), the Chongqing Philosophy and Social Science Development Programs (2021NDYB052), and the Scientific Research Programs of Southwest University of Political Science and Law (2020XZYB-11).

## Conflict of interest

The authors declare that the research was conducted in the absence of any commercial or financial relationships that could be construed as a potential conflict of interest.

## Publisher’s note

All claims expressed in this article are solely those of the authors and do not necessarily represent those of their affiliated organizations, or those of the publisher, the editors and the reviewers. Any product that may be evaluated in this article, or claim that may be made by its manufacturer, is not guaranteed or endorsed by the publisher.

## References

[ref1] Angelovski A. di Cagno D. Grieco D. Güth W. (2019). Trusting versus monitoring: an experiment of endogenous institutional choices. Evolut. Institut. Econ. Rev. 16, 329–355. doi: 10.1007/s40844-019-00126-4

[ref2] Anwar S. Sun S. (2015). Unionisation and firm performance in China’s manufacturing industries. J. Lab. Res. 36, 78–102. doi: 10.1007/s12122-014-9197-1

[ref3] Balliet D. Van Lange P. A. M. (2013). Trust, conflict, and cooperation: A meta-analysis. Psychol. Bull. 139, 1090–1112. doi: 10.1037/a0030939, PMID: 23231532

[ref4] Barrett R. (1995). Factors affecting perceptions of a workplace industrial relations climate. Int. J. Employ. Stud. 3, 77–90.

[ref5] Buchanan J. (2008). Inside the workplace - findings from the 2004 workplace employment relations survey - By Barbara Kersley, Carmen Alpin, John forth, Alex Bryson, Helen Bewley, gill dix and Sarah Oxenbridge. Br. J. Ind. Relat. 46, 565–567. doi: 10.1111/j.1467-8543.2008.00690_6.x

[ref6] Budd J. W. Lamare J. R. (2021). The importance of political Systems for Trade Union Membership, coverage and influence: theory and comparative evidence. Br. J. Ind. Relat. 59, 757–787. doi: 10.1111/bjir.12575

[ref7] Butler J. K. (1991). Toward understanding and measuring conditions of trust: evolution of a conditions of trust inventory. J. Manag. 17, 643–663. doi: 10.1177/014920639101700307

[ref8] Chan C. K. C. Hui E. S. (2018). The dynamics and dilemma of workplace trade union reform in China: The case of the Honda workers’ strike. J. Ind. Relat. 60:717. doi: 10.1177/0022185612457128

[ref9] Chan A. W. Snape E. Luo M. S. Zhai Y. (2017). The developing role of unions in China’s foreign-invested enterprises: unions in China’s foreign-invested enterprises. Br. J. Ind. Relat. 55, 602–625. doi: 10.1111/bjir.12218

[ref10] Chan A. W. Snape E. Redman T. (2004). Union commitment and participation among Hong Kong firefighters: a development of an integrative model. Int. J. Hum. Resour. Manag. 15, 533–548. doi: 10.1080/0958519042000181241

[ref11] Chen F. (2010). Trade unions and the quadripartite interactions in strike settlement in China. China Q. 201, 104–124. doi: 10.1017/S0305741009991093

[ref12] Chung S. W. (2016). Industrial relations (IR) changes in China: a foreign employer’s perspective. Empl. Relat. 38, 826–840. doi: 10.1108/ER-06-2015-0120

[ref13] Cooke F. L. (2014). Chinese industrial relations research: In search of a broader analytical framework and representation. Asia Pac. J. Manag. 31, 875–898. doi: 10.1007/s10490-014-9386-8

[ref14] Cui X. Zhang Y. Qu J. (2012). Labour relations climate and job satisfaction: the moderating role of organizational commitment. Nankai Bus. Rev. 15, 19–30.

[ref15] Dastmalchian A. Blyton P. Adamson R. (1989). Industrial-relations climate- testing a construct. J. Occup. Psychol. 62, 21–32. doi: 10.1111/j.2044-8325.1989.tb00474.x

[ref16] Ehlers L. I. (2020). Trust and perceptions of compliance, fairness and good faith in primary labour relationships. S. Afr. J. Econ. Manag. Sci. 23:a3353. doi: 10.4102/sajems.v23i1.3353

[ref17] Ellonen R. Blomqvist K. Puumalainen K. (2008). The role of trust in organisational innovativeness. Eur. J. Innov. Manag. 11, 160–181. doi: 10.1108/14601060810869848

[ref18] Farrell H. Knight J. (2003). Trust, institutions, and institutional change: industrial districts and the social capital hypothesis. Polit. Soc. 31, 537–566. doi: 10.1177/0032329203256954

[ref19] Friedman E. (2012). Getting Through the hard times together? Chinese workers and unions respond to the economic crisis. J. Ind. Relat. 54, 459–475. doi: 10.1177/0022185612448762

[ref20] Fuller J. B. Hester K. (1998). The effect of labor relations climate on the union participation process. J. Lab. Res. 19, 173–187. doi: 10.1007/s12122-998-1009-z

[ref21] Furaker B. (2020). European trade union cooperation, union density and employee attitudes to unions. Transfer 26, 345–358. doi: 10.1177/1024258920933118

[ref22] Ge Y. (2014). Do CHINESE unions have “real” effects on employee compensation? Contemp. Econ. Policy 32, 187–202. doi: 10.1111/coep.12012

[ref23] Guo R. Laroche P. (2021). The effects of trade unions on economic performance: evidence from Chinese provincial-level data. Int. J. Manpow. 42, 1084–1101. doi: 10.1108/IJM-11-2018-0369

[ref24] Heng H. (2010). Concerns About the trade union System’s independent interests: survey and thoughts regarding the trade unions at grass-roots enterprises. Chin. Sociol. Anthropol. 42, 61–73. doi: 10.2753/CSA0009-4625420304

[ref25] Hu E. Zhang M. Shan H. Zhang L. Yue Y. (2018). Job satisfaction and union participation in China: developing and testing a mediated moderation model. Empl. Relat. 40, 964–980. doi: 10.1108/ER-10-2017-0245

[ref26] Huang W. Li Y. Wang S. Weng J. (2016). Can ‘democratic management’ improve labour relations in market-driven China? Asia Pac. J. Hum. Resour. 54, 230–257. doi: 10.1111/1744-7941.12100

[ref27] Huang W. Yuan C. Shen J. Li M. (2021). Effects of union commitment on job performance in China. Pers. Rev. 50, 1185–1199. doi: 10.1108/PR-06-2019-0323

[ref28] Innocenti L. Pilati M. Peluso A. M. (2011). Trust as moderator in the relationship between HRM practices and employee attitudes. Hum. Resour. Manag. J. 21, 303–317. doi: 10.1111/j.1748-8583.2010.00151.x

[ref29] Jiang Y. Yao Y. (2020). Industrial relations climate and employee intention to quit: The roles of voice and silence. Soc. Behav. Personal. Int. J. 48, 1–13. doi: 10.2224/sbp.9235

[ref30] Jones A. P. James L. R. (1979). Psychological climate: dimensions and relationships of individual and aggregated work environment perceptions. Organ. Behav. Hum. Perform. 23, 201–250. doi: 10.1016/0030-5073(79)90056-4

[ref31] Kai C. Brown W. (2013). The transition from individual to collective labour relations in China. Ind. Relat. J. 44, 102–121. doi: 10.1111/irj.12013

[ref32] Kougiannou K. Redman T. Dietz G. (2015). The outcomes of works councils: the role of trust, justice and industrial relations climate. Hum. Resour. Manag. J. 25, 458–477. doi: 10.1111/1748-8583.12075

[ref33] Lee C.-H. Brown W. Wen X. (2016). What Sort of collective bargaining is emerging in China? Br. J. Ind. Relat. 54, 214–236. doi: 10.1111/bjir.12109

[ref34] Lewicka D. (2020). Employee institutional trust as an antecedent of diverse dimensions of Organisational commitment. Argum. Oeconom. 2019, 321–340. doi: 10.15611/aoe.2020.1.13

[ref35] Liu M. Li C. (2014). Environment pressures, managerial industrial relations ideologies and unionization in Chinese enterprises: determinants of unionization in Chinese enterprises. Br. J. Ind. Relat. 52, 82–111. doi: 10.1111/j.1467-8543.2012.00908.x

[ref36] Luethje B. (2014). Labour relations, production regimes and labour conflicts in the Chinese automotive industry. Int. Labour Rev. 153, 535–560. doi: 10.1111/j.1564-913X.2014.00215.x

[ref37] Mayer R. C. Davis J. H. Schoorman F. D. (1995). An integrative model of organizational trust. Acad. Manag. Rev. 20:709. doi: 10.2307/258792

[ref38] Mellor S. Golay L. M. (2014). The conditional indirect effect model of Women’s union participation: The moderating effect of perceived union tolerance for sexual harassment. Aust. J. Psychol. 148, 73–91. doi: 10.1080/00223980.2012.748580, PMID: 24617272

[ref39] Murphy R. (2020). Why unions survive: understanding how unions overcome the free-rider problem. J. Labor Econ. 38, 1141–1188. doi: 10.1086/706091

[ref40] Newman A. Cooper B. Holland P. Miao Q. Teicher J. (2019). How do industrial relations climate and union instrumentality enhance employee performance? The mediating effects of perceived job security and trust in management. Hum. Resour. Manag. 58, 35–44. doi: 10.1002/hrm.21921

[ref41] Ocasio W. Radoynovska N. (2016). Strategy and commitments to institutional logics: organizational heterogeneity in business models and governance. Strateg. Organ. 14, 287–309. doi: 10.1177/1476127015625040

[ref42] Payne R. Mansfield R. (1978). Correlates of individual perceptions of organizational climate. J. Occup. Psychol. 51, 209–218. doi: 10.1111/j.2044-8325.1978.tb00417.x

[ref43] Pyman A. Holland P. Teicher J. Cooper B. K. (2010). Industrial relations climate, employee voice and managerial attitudes to unions: an Australian study. Br. J. Ind. Relat. 48, 460–480. doi: 10.1111/j.1467-8543.2009.00772.x

[ref44] Redman T. Snape E. (2014). The antecedents of union commitment and participation: evaluating moderation effects across unions. Ind. Relat. J. 45, 486–506. doi: 10.1111/irj.12073

[ref45] Refslund B. (2021). When strong unions meet precarious migrants: building trustful relations to unionise labour migrants in a high union-density setting. Econ. Ind. Democr. 42, 314–335. doi: 10.1177/0143831X18760989

[ref46] Ringqvist J. (2021). How do union membership, union density and institutionalization affect perceptions of conflict between management and workers? Eur. J. Ind. Relat. 27, 131–148. doi: 10.1177/0959680120963546

[ref47] Schneider B. Reichers A. E. (1983). On the etiology of climates. Pers. Psychol. 36, 19–39. doi: 10.1111/j.1744-6570.1983.tb00500.x

[ref48] Shapiro S. P. (1987). The social control of impersonal trust. Am. J. Sociol. 93, 623–658. doi: 10.1086/228791

[ref49] Snape E. Redman T. (2012). Industrial relations climate and union commitment: An evaluation of workplace-level effects. Indus. Relat. 51, 11–28. doi: 10.1111/j.1468-232X.2011.00662.x

[ref50] Sverke M. Kuruvilla S. (1995). A new conceptualization of union commitment: development and test of an integrated theory. J. Organ. Behav. 16, 505–532. doi: 10.1002/job.4030160603

[ref51] Tyler T. R. (2003). Trust within organisations. Pers. Rev. 32, 556–568. doi: 10.1108/00483480310488333

[ref52] Valizade D. (2018). A new theory of industrial relations: people, markets and organizations after neoliberalism. Br. J. Ind. Relat. 56, 686–688. doi: 10.1111/bjir.12423

[ref53] Vanhala M. Puumalainen K. Blomqvist K. (2011). Impersonal trust: The development of the construct and the scale. Pers. Rev. 40, 485–513. doi: 10.1108/00483481111133354

[ref54] Wei Q. Hu E. Sun J.. (2021). Double-edged sword effect of high-performance work system on employee well-being—moderating effect of union practice. Front. Psychol. 12:619345. doi: 10.3389/fpsyg.2021.619345, PMID: 34421698 PMC8374043

[ref55] Wu Q. Sun Z. (2014). Collective consultation under quota management: China’s government-led model of labour relations regulation. Int. Labour Rev. 153, 609–633. doi: 10.1111/j.1564-913X.2014.00218.x

[ref56] Xi M. Zhou L. Zhang X. Zhao S. (2021). Labor relations conflict in China: An analysis of conflict measure, conflict solution and conflict outcomes. Int. J. Hum. Resour. Manag. 33, 1–37. doi: 10.1080/09585192.2021.1903966

[ref57] Xie P. Zhou L. (2020). Keeping dispute resolution internal: exploring the role of the industrial relations climate, organizational embeddedness and organizational turbulence. Econ. Ind. Democr. 43, 899–917.

[ref58] Yao Y. Zhong N. (2013). Unions and workers’ welfare in Chinese firms. J. Labor Econ. 31, 633–667. doi: 10.1086/669819

[ref59] Yuan L. Yu Y. Li J. Ning L. (2014). Occupational commitment, industrial relations and turnover intention empirical evidence from China. Chin. Manag. Stud. 8, 66–84. doi: 10.1108/CMS-08-2011-0065

[ref60] Zhang M. Hu E. Zhang L. (2018). Enterprise Union practice in China: development and validation of a measurement scale. Bus. manag. J. 40, 39–54. doi: 10.19616/j.cnki.bmj.2018.11.003

[ref61] Zhu Y. Warner M. Feng T. (2011). Employment relations “with Chinese characteristics”: The role of trade unions in China. Int. Labour Rev. 150, 127–143. doi: 10.1111/j.1564-913X.2011.00108.x

[ref62] Zhu J. S. Zhu C. J. (2022). Culture lag in Chinese labour relations: managers’ perceptions and behaviour towards workplace trade unions (2009 - 2014). Labor History 63, 24–36. doi: 10.1080/0023656X.2022.2040458

